# Miliary Tuberculosis Presenting with ARDS and Shock: A Case Report and Challenges in Current Management and Diagnosis

**DOI:** 10.1155/2017/9287021

**Published:** 2017-11-28

**Authors:** Keevan Singh, Saara Hyatali, Stanley Giddings, Kevin Singh, Neil Bhagwandass

**Affiliations:** ^1^Anaesthesia and Intensive Care Department, San Fernando General Hospital and Department of Clinical Surgical Sciences, University of the West Indies, San Fernando, Trinidad, Trinidad and Tobago; ^2^Internal Medicine Department, San Fernando General Hospital, San Fernando, Trinidad and Tobago; ^3^Infectious Disease Unit, San Fernando General Hospital and Department of Clinical Medical Sciences, University of the West Indies, San Fernando, Trinidad, Trinidad and Tobago

## Abstract

Miliary tuberculosis, complicated by ARDS and septic shock, is a rare and lethal presentation of this disease. Here we present a case of such a patient, following which we discuss the management of tuberculosis in the ICU and some of the challenges that may be faced. A young HIV negative female presented to us with an acute history of worsening shortness of breath on a background of weight loss, nonproductive cough, and fever. CXR and CT scan showed bilateral miliary type opacities and the patient was admitted to the hospital. Within forty-eight hours of admission she became hypoxemic and was intubated and transferred to the ICU. There she experienced worsening organ dysfunction and developed circulatory shock. Despite escalating doses of noradrenaline, she continued to decline and died before specific anti-TB treatment could be started. Timely diagnosis and treatment initiation are the keys to improving outcomes in critically ill TB patients. However there are many challenges in doing so, especially in a general ICU located in a country with a low TB incidence.

## 1. Introduction

Globally, there has been a decline in the incidence as well as number of tuberculosis (TB) deaths [1]. However the disease continues to have a significant burden with more than ten million cases and one million deaths recorded in 2015 [1]. The Southeast Asian Region bears a large part of this disease burden, with significantly lower disease rates recorded in many western countries [1, 2].

Trinidad and Tobago lying in the Southern Caribbean has an incidence of approximately 17 cases per 100 000 persons [3]. With this relatively low incidence rate, tuberculosis is seldom seriously considered among the primary diagnoses, especially in the setting of ARDS and shock where other diagnoses are more likely.

Although outcome data shows an 83% success rate for TB treatment, presentation to an ICU setting with multiorgan failure can carry an extremely poor prognosis [1, 4, 5]. Delayed diagnosis and treatment initiation due to lack of recognition in low incidence countries are some of the chief contributors to this mortality [4, 6].

The following case illustrates this in the rapid decline of a young HIV negative female with miliary TB who presented to us with acute respiratory failure. The patient's condition progressed quickly to fatal circulatory shock despite supportive ICU care. Following the case, we also discuss the prognosis and current ICU management and challenges in identifying and managing a critically ill TB patient.

## 2. Case Report

A 16-year-old female was admitted to hospital with a one-week history of gradually worsening shortness of breath. For more than two months, she described having a nonproductive cough, nocturnal fever, significant weight loss, joint pains, and fatigue.

The patient had previously sought treatment from her local primary care physician for a cough and fever approximately two months prior to this presentation. At this time, a HIV test was done which was negative. No further follow-up was done until her subsequent deterioration and presentation at our hospital.

On presentation the patient was tachypnoeic and tachycardic, with a respiratory rate of 40 bpm, a heart rate of 148 bpm, and a temperature of 37.8°C. Her oxygen saturation was 100% on facemask oxygen at 6 L/min with a blood pressure of 123/85 mmHg. Crepitations were heard throughout both lung fields. The remainder of the physical exam was normal with the patient noted to be alert and oriented.

**Figure 1:**
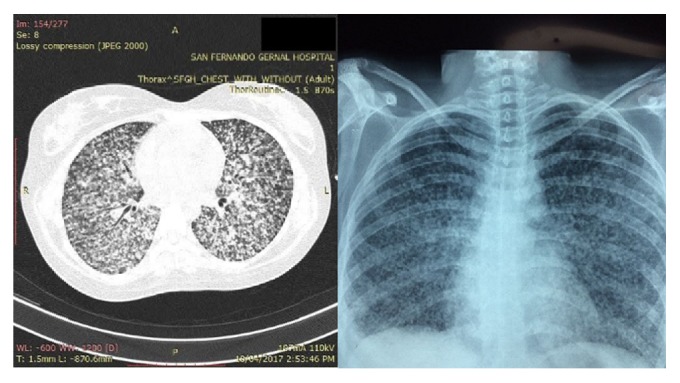
CT scan and CXR of patient showing bilateral miliary opacification.

An urgent CXR was requested which showed multiple miliary opacities scattered throughout both lung fields (Figure 1). Laboratory results at this time were significant for microcytic anemia, leukocytosis (Hb, 7.6 g/dL, MCV, 76 fL, and WBC, 15.4 × 10^9^/L) and a normal platelet count (332 × 10^9^/L). Serum creatinine and electrolytes were grossly normal. A CT scan of the chest confirmed miliary opacities, with no mediastinal or hilar lymphadenopathy (Figure 1).

Differential diagnoses at this time included pneumocystis pneumonia, viral/fungal pneumonia, miliary tuberculosis, and metastatic disease. Patient was reviewed by the Infectious Disease Unit and transferred to an isolation room on the medical ward.

An HIV rapid test was performed, which was subsequently reported as negative [Determine™ and UNI-GOLD™]. Due to the lack of any historical evidence suggesting frequent infections or an immunocompromised state, no immunological tests were requested. An interferon-gamma release assay [QuantiFERON™] was also requested and the patient was empirically started on levofloxacin, fluconazole, and acyclovir.

On day 2 of admission the patient deteriorated (SPO2 70% on Nonrebreather Mask set at 15 L/min), was transferred to the ICU, intubated, and placed on mechanical ventilation. Her P/F ratio at that time was 130 indicating moderate ARDS. After initial stabilization, worsening oxygenation (P/F ratio 113) prompted administration of bolus and infusion of cisatracurium. The patient was ventilated using a lung protective approach with a tidal volume of 330 ml (6 ml/kg), a PEEP of 10 cmH2O, RR of 20, and flow rate of 35 L/min. Sputum, via endotracheal tube suction catheter, was taken for testing at the regional public health lab. On a FiO2 setting ranging from 70 to 100%, the patient's oxygen saturation remained between 85% and 90%.

On further questioning of the patient's parents, it was revealed that she was in contact with a relative who had a nonproductive cough of unknown but prolonged duration. There was no testing or TB confirmation done in the family.

On admission day 3, patient was noted to be hypotensive with MAP's < 60 mmHg and persistently pyretic. Multiple polygeline fluid boluses were given and a noradrenaline infusion was started. As her condition continued to deteriorate, the noradrenaline dose increased to a max of 50 ug/min. Despite this the patient's hypotension persisted and she progressed to a fatal cardiac arrest early on the fourth day.

Shortly after her demise, Nucleic Acid Amplification [Xpert™ MTB/RIF] results from her sputum became available and it was positive for* Mycobacterium tuberculosis* with no rifampin resistance being detected. No smear microscopy or culture was performed in this case. Previously requested QuantiFERON test was marred by postprocessing issues and a retest was suggested.

On the evening before her death, a decision had been made to empirically start antituberculosis therapy; however due to its infrequent presentation in our regional hospital, no drugs were kept in stock and they had to be sourced from our local TB treatment center. Unfortunately, the patient's death preempted this.

## 3. Discussion

### 3.1. Prognosis

Miliary TB is described as a form of disseminated TB that results from lymphatic and hematologic spread from a TB focus and is invariably fatal if left untreated [8]. Recent data from countries with a low to moderate incidence of TB shows a 14% mortality in treated patients with miliary TB [9]. However, mortality increases once ARDS and septic shock and multiorgan failure develop.

In a cohort of 469 ARDS patients, from a high incidence setting, only 3.6% of the cases were due to* Mycobacterium tuberculosis* (MTB) [10]. Similarly the incidence of MTB as the primary cause of septic shock was less than one percent in a large septic shock cohort from low-moderate incidence countries [4]. Although the incidence of both ARDS and septic shock secondary to MTB is quite low in the general cohort of ICU patients, patients with TB needing ICU admission can have a > 90% rate of developing ARDS [5].

Kethireddy et al. showed that in patients with MTB septic shock the mortality rate was 79% (versus 49% in those with non-MTB septic shock) and only one patient who received anti-TB therapy after 24 hours survived [4]. In patients with ARDS secondary to MTB, recent data has shown a similar mortality rate to nontuberculous ARDS; however older studies have indicated mortality rates of 60–88% for MTB ARDS [10–12].

Overall, quoted ICU mortality can range from 26 to 66% for patients admitted with TB [5, 6, 13]. Mortality predictors included delayed treatment initiation by >1 month, baseline organ dysfunction, and number of organ failures [6, 10].

### 3.2. Diagnosis

In low incidence countries, miliary TB presenting as ARDS or septic shock can be easily missed, especially given the scarcity of these presentations in HIV negative patients even in endemic regions [4, 10]. However, given the high mortality rate associated with delayed treatment, it is important that the diagnosis be confirmed early so that treatment can be started promptly.

In addition to historical features, which can be variable, miliary type opacifications may be found in more than 80% of patients with miliary TB [9, 14]. As such, finding miliary opacifications should prompt rapid and further diagnostic testing so as to facilitate early treatment initiation. Other causes of miliary opacification are listed in Box 1.

Diagnosis of TB can be time consuming and challenging. This is especially true in the ICU patient where a delayed diagnosis can prove fatal and an appropriate specimen can be difficult to obtain. Standard diagnostic testing involves AFB smear microscopy followed by liquid and solid mycobacterial culture [15]. Mycobacterial culture can take up to a month to be available, while molecular techniques, as was done in this case, can give a result in hours and is recommended by consensus guidelines [15, 16]. In the critically ill patient where a high degree of clinical suspicion exists for disseminated or pulmonary TB, consideration should be given to obtain a sputum sample urgently for molecular testing and drug sensitivity.

### 3.3. ICU Management

High quality, supportive ICU care is essential in managing the TB patient with organ dysfunction. In patients with miliary TB presenting with ARDS and respiratory failure, particular attention should be paid to low tidal volume ventilation, conservative fluid administration, early use of neuromuscular blocking agents, and prone positioning in severe cases [16]. Of note the increased risk of pneumothorax in pulmonary TB would prompt the clinician to be vigilant in cases where high PEEP is used [17]. It is possible that the lower mortality rates seen in the recent work by Muthu et al. represent the effect of some of these changes [10].

In patients presenting with tuberculous septic shock, as in our case, standard management for bacterial shock should be followed. The core management principles involve early antibiotic use, fluid administration, and vasopressors to correct persisting hypotension—as described in the early goal directed protocol by Rivers et al. [18]. Although current evidence does not support a rigid application of this early goal directed protocol, the core principles in the management of septic shock remain the same [19].

Initiating anti-TB therapy can be challenging in the ICU patient. Issues include poor oral absorption, lack of parenteral formulations for some agents, and the presence of renal and hepatic dysfunction which is commonly seen in the critically ill patient. Standard therapy for TB involves a combination of three or more agents and usually contains isoniazid and rifampicin both of which are hepatotoxic [20]. Early consultation with an infectious disease specialist can help guide effective therapy. Additional options include the use of Therapeutic Drug Monitoring when oral dosing is used in critically ill patients, especially when delayed gastric emptying is an issue, and use of parenteral fluoroquinolones and aminoglycosides when liver dysfunction is a concern [20].

### 3.4. Challenges in the ICU

Despite efforts to contain and minimize the spread of contagious diseases in the ICU, several challenges remain in isolating and nursing such patients. Tuberculosis, though of low incidence, is no exception.

The main mode of transmission of TB is via droplet nuclei, dispersal of which is very common in the ICU setting during endotracheal intubation, suctioning, bronchoscopy, sputum induction, and administration of aerosolized medication [21]. According to the CDC, at least an N95 level of airway protection should be used for any healthcare worker entering a room with an infectious TB patient and especially during any of these procedures [21]. Consideration may be made for higher levels of respiratory protection during bronchoscopy as there is a higher risk of transmission. N95 masks are usually available in most healthcare settings; however there is often a lack of PAPRs (powered air-purifying respirators) if further protection is needed.

Most ICUs are designed with a fixed number of negative pressure isolation rooms. The number of these rooms is dependent on the function and case mix of the ICU [22]. In ICUs that deal with a low number of highly infectious cases, isolation room numbers may be quite small, whereas negative pressure rooms situated outside of the ICU are inadequate to deal with a critically ill patient. Such rooms are the standard of care for housing hospitalized patients with active pulmonary TB [21].

In many low incidence countries TB is treated in specialized centers. As a result, many smaller hospitals and ICUs may not have easy access to rapid confirmatory testing. Also, oral and parenteral TB drugs may not be available in a general ICU in these settings. All of these contribute to delays which may prove fatal in the critically ill patient.

## 4. Conclusion

ARDS and septic shock are rare presentations of miliary TB even in countries with a high TB incidence. Our case describes a patient who presented with both shock and respiratory failure and her subsequent fatal course. In addition to organ support, these patients need early definitive anti-TB treatment. Miliary shadowing observed on imaging a critically ill patient with organ failure may warrant molecular methods in testing for TB, so as to facilitate prompt treatment. Availability of parenteral anti-TB drugs and droplet transmission and availability of negative pressure isolation rooms are further challenges that can be faced, especially in smaller ICUs.

## Figures and Tables

**Box 1:**
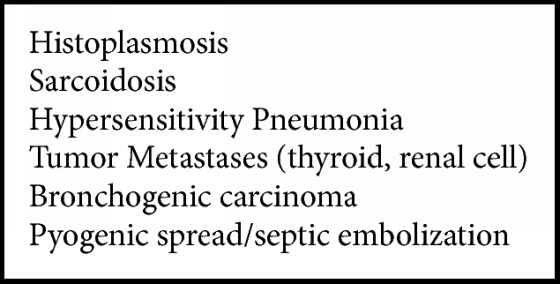
Causes of miliary type opacifications. Modified from [7].

## Data Access

Data is available on request to the corresponding author.
