# Corrigendum to “Miliary Tuberculosis Presenting with ARDS and Shock: A Case Report and Challenges in Current Management and Diagnosis”

**DOI:** 10.1155/2018/2073618

**Published:** 2018-05-14

**Authors:** Keevan Singh, Saara Hyatali, Stanley Giddings, Kevin Singh, Neal Bhagwandass

**Affiliations:** ^1^Anaesthesia and Intensive Care Department, San Fernando General Hospital and Department of Clinical Surgical Sciences, University of the West Indies, San Fernando, Trinidad, Trinidad and Tobago; ^2^Internal Medicine Department, San Fernando General Hospital, San Fernando, Trinidad and Tobago; ^3^Infectious Disease Unit, San Fernando General Hospital and Department of Clinical Medical Sciences, University of the West Indies, San Fernando, Trinidad, Trinidad and Tobago

In the article titled “Miliary Tuberculosis Presenting with ARDS and Shock: A Case Report and Challenges in Current Management and Diagnosis” [1], the name of the fifth author was given incorrectly as Neil Bhagwandass. The author's name should have been written as Neal Bhagwandass. The revised authors' list is shown above.
